# Is the prediction of prognosis not improved by the seventh edition of the TNM classification for colorectal cancer? Analysis of the surveilla006Ece, epidemiology, and end results (SEER) database

**DOI:** 10.1186/1471-2407-13-123

**Published:** 2013-03-17

**Authors:** Peng Gao, Yong-xi Song, Zhen-ning Wang, Ying-ying Xu, Lin-lin Tong, Jing-xu Sun, Miao Yu, Hui-mian Xu

**Affiliations:** 1 Department of Surgical Oncology and General Surgery, the First Hospital of China Medical University, Shenyang City, 110001, PR China

**Keywords:** TNM system, Colorectal cancer, Prognostic prediction

## Abstract

**Background:**

Whether the 7th edition of American Joint Committee on Cancer (AJCC) TNM staging system (AJCC-7) is a successful revision remains debatable. We aimed to compare the predictive capacity of the AJCC-7 for colorectal cancer with the 6th edition of the AJCC TNM staging system (AJCC-6).

**Methods:**

The National Cancer Institute’s Surveillance, Epidemiology, and End Results (SEER) dataset consisting of 158,483 records was used in this study. We evaluated the predictive capacity of the two editions of the staging system using Harrell’s C index and Bayesian Information Criterion (BIC).

**Results:**

There was a significant prognostic difference between patients at stage IIB and IIC (P < 0.001). Stage III patients with similar prognoses were adequately sub-grouped in the same stage according to AJCC-7. The Harrell’s C index revealed a value of 0.7692 for AJCC-7, which was significantly better than 0.7663 for AJCC-6 (P < 0.001). BIC analysis provided consistent results (P < 0.001).

**Conclusions:**

This study demonstrates that AJCC-7 is superior to the AJCC-6 staging system in predictive capacity.

## Background

Colorectal cancer is one of the most common malignancies worldwide [1]. Accurate prognostic prediction of patients with colorectal cancer is essential for improved treatment selection. The cancer stage is the strongest predictor of survival for patients with colorectal cancer. Accurate staging enables physicians to stratify patients in terms of expected predicted survival in order to help select the most effective treatments, determine prognoses, and evaluate cancer control measures [2]. The International American Joint Committee on Cancer (AJCC) TNM staging system is currently regarded as the strongest prognostic parameter for patients with colorectal cancer [3]. Over the past several decades, the TNM staging system has continued to develop, and in 2010 the 7th revision of TNM staging [4] (AJCC-7) was published by the AJCC and replaced the 6th edition [5] (AJCC-6) issued in 2002. The basic staging principals remained unchanged; however, some subtle changes were made. The major changes in the AJCC-7 are as follows:

1) T4 lesions are subdivided as T4a (Tumor penetrates the surface of the visceral peritoneum) or T4b. (Tumor directly invades or is histologically adherent to other organs or structures);

2) T1-2 lesions that lack regional lymph node metastasis but exhibit tumor deposit(s) are classified in addition as N1c;

3) N1 tumors are subdivided as N1a (metastasis in 1 regional node) and N1b (metastasis in 2–3 nodes), and N2 tumors are subdivided as N2a (metastasis in 4–6 nodes) and N2b (metastasis in 7 or more nodes);

4) Stage Group II is subdivided into IIA (T3N0), IIB (T4aN0), and IIC (T4bN0);

5) T4bN1 is reclassified from IIIB to IIIC;

6) T1N2a is reclassified as IIIA, and T1N2b, T2N2a-b, and T3N2a are all reclassified as IIIB;

7) M1 tumors are subdivided into M1a (single metastatic site) and M1b (multiple metastatic sites).

**Figure 1:**
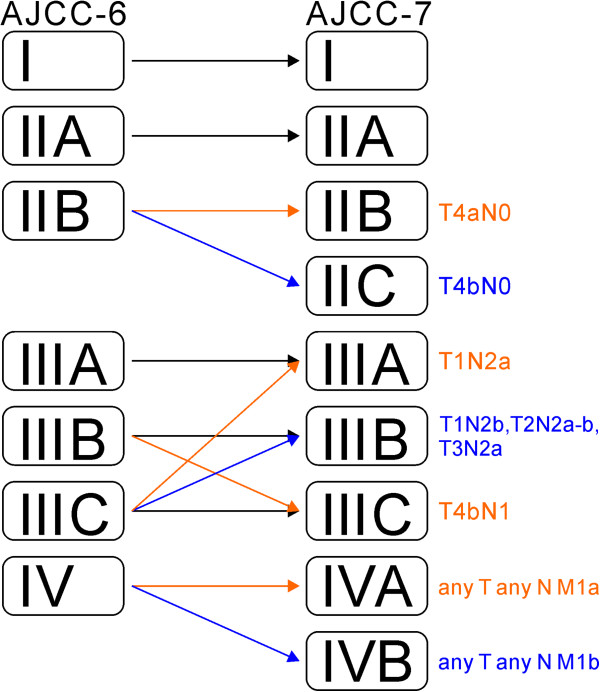
Diagram of the major differences between the 6th and the 7th editions of the American Joint Committee on Cancer (AJCC-6 and AJCC-7, respectively) staging system for colorectal cancer.

A diagram of the major differences between AJCC-7 and AJCC-6 was presented in Figure 1.

After the publication of AJCC-7, its prognostic validity was discussed worldwide. Whether AJCC-7 is a successful revision remains debatable. Some previous studies have validated supported the revisions introduced [6-9]. However, reports by Mori [10] and Nagtegaal [11,12] have raised objections to the revisions; in particular, Nitsche [13] suggested that AJCC-7 did not provide greater accuracy in predicting colorectal cancer patients’ prognosis compared with AJCC-6.

This study aimed to analyze the prognostic validity of AJCC-7 for colorectal cancer in comparison to AJCC-6; we applied the National Cancer Institute’s Surveillance, Epidemiology, and End Results (SEER) dataset for statistical analysis.

## Methods

### Data from the SEER program

The dataset we used is the National Cancer Institute’s SEER dataset, 1973–2008. SEER collects data on cancer cases from various locations and sources throughout the United States and the program is regarded as a model population-based tumor registry. This national program includes 17 regional registries that cover approximately 28% of the US population. The number of records included in the SEER dataset reaches 6,551,087, including 5,937,405 malignant cases. Among these patients, more than 670,000 patients suffered from colorectal cancer. Patients diagnosed from 1991 through 2003 were selected for analysis. The primary study endpoint was cancer-specific survival.

We selected tumors according to the primary site as follows: cecum, ascending colon, hepatic flexure, transverse colon, splenic flexure, descending colon, sigmoid colon, overlapping lesion of colon, colon NOS (not otherwise specified), rectosigmoid junction, and rectum NOS. We further restricted the tumors included by specific histologic type, as defined by the following individual International Classification of Diseases for Oncology, third edition (ICD-O-3), codes: 8000–8152, 8154–8231, 8243–8245, 8250–8576, 8940–8950, 8980–8981 in accordance with AJCC-7 [4]. Patients were excluded from this study if they exhibited: 1) prior non-colorectal cancer; 2) *in situ* tumors; 3) incomplete pathological data entries; or 4) died during the immediate postoperative period (within 30 days).

After using these inclusion and exclusion strategies, a dataset consisting of 158,483 records was constructed and the following data were recorded: age, gender, race, primary tumor site, number of lymph nodes retrieved, AJCC-6 TNM stage, and AJCC-7 TNM stage. Both TNM stages were determined by SEER’s “extent of disease” (for T category and M category) and “regional nodes positive” (for N category) coding schemes. The N1c category was not included because the information of tumor deposits was not supported by the SEER program. We considered stage IV in its entirety because the number of metastatic organ/site was unknown in the SEER program. The clinicopathologic features of the colorectal patients are listed in Table 1.

**Table 1:** Clinicopathologic features of the colorectal cancer patients

	**n**	**%**
Gender		
Male	79272	50.0
Female	79211	50.0
Age*	68.45 ± 13.07	
Race		
White	130774	82.5
Black	14252	9.0
Other	13457	8.5
Tumor location		
Colon	119903	75.6
Rectum	38580	24.4
Histologic grade		
Well	13031	8.2
Moderate	112228	70.8
Poor	32108	20.3
Undifferentiated	1116	0.7
Number of lymph nodes retrieved*	12.10 ± 8.87	
pT category∫		
pT1	14149	8.9
pT2	24573	15.5
pT3	81507	51.4
pT4a	9909	6.3
pT4b	6982	4.4
pN category		
pN0	91722	57.9
pN1a	19449	12.3
pN1b	21198	13.4
pN2a	14356	9.1
pN2b	11758	7.4
pM category		
pM0	137120	86.5
pM1	21363	13.5

*: Mean ± SD.

∫: The pT categ.ory of patients with distant metastasis is unavailable.

### Ethics statement

This study was in compliance with the Helsinki Declaration. We have got permission to access the research data file in SEER program and the reference number was 10188-Nov2011. The study was also approved by the Research Ethics Committee of China Medical University, China and the reference number was [2012]96.

### Statistical analysis

Continuous data are presented as mean ± standard deviation (SD). Cancer-specific survival was analyzed using Kaplan-Meier survival curves, and comparisons were made using the log-rank test.

We evaluated the predictive capacity of the different categories by investigating measures of discrimination. Discrimination refers to the ability to distinguish between high-risk and low-risk patients, and we quantified discrimination using the Harrell’s C index [14,15] and the Bayesian Information Criterion (BIC) [16]. A model with perfect predictive capacity (sensitivity and specificity of 100%) would have a Harrell’s c-index of 1.00; a category that exhibited a higher Harrell’s C index was considered to exhibit a more accurate predictive capacity. BIC was used to assess the overall prognostic performance of different classification systems via bootstrap-resampling analysis. A smaller BIC value indicates a more desirable model for predicting outcome.

All statistical analyses and graphics were performed using the PASW Statistics 18.0 software (SPSS, Inc., Somers, NY, USA) and STATA MP ver. 10 (StataCorp LP, College Station, TX) statistical software. For all analyses, P < 0.05 was considered to indicate a significant result.

## Results

### Survival outcomes by two editions of staging system

**Figure 2:**
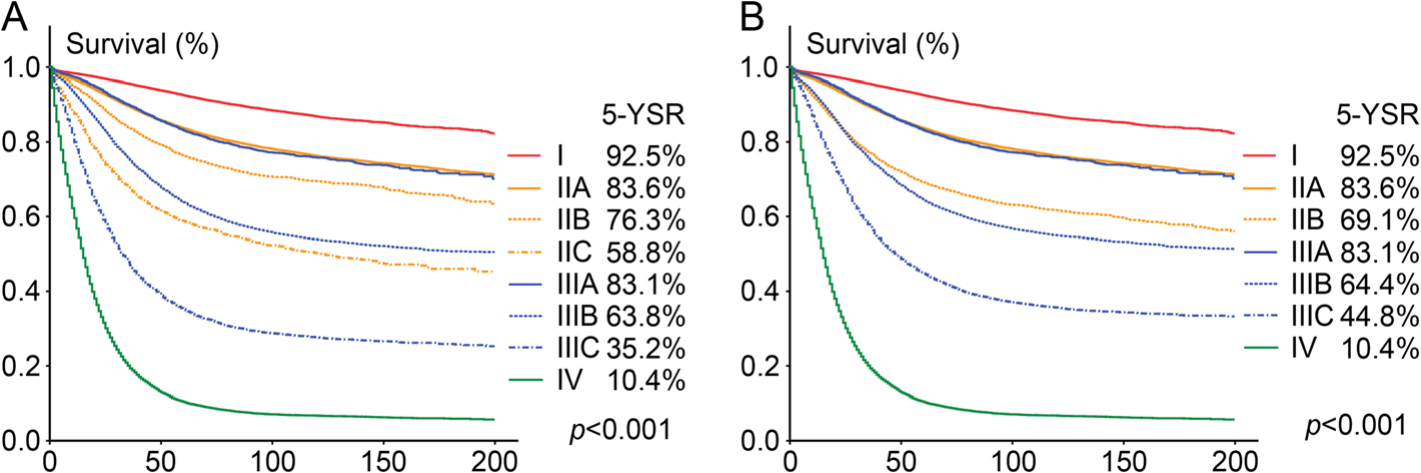
**Survival curves of patients in two datasets.** Stage-dependent survival according to allocation of the patients to (**a**), seventh, or (**b**), sixth edition of the TNM classification.

Comparisons of survival curves between patients in different TNM stage according to the AJCC-6 and AJCC-7 were presented in Figure 2. As shown, significant prognostic differences could always be observed in both AJCC-6 and AJCC-7 (p < 0.001).

**Figure 3:**
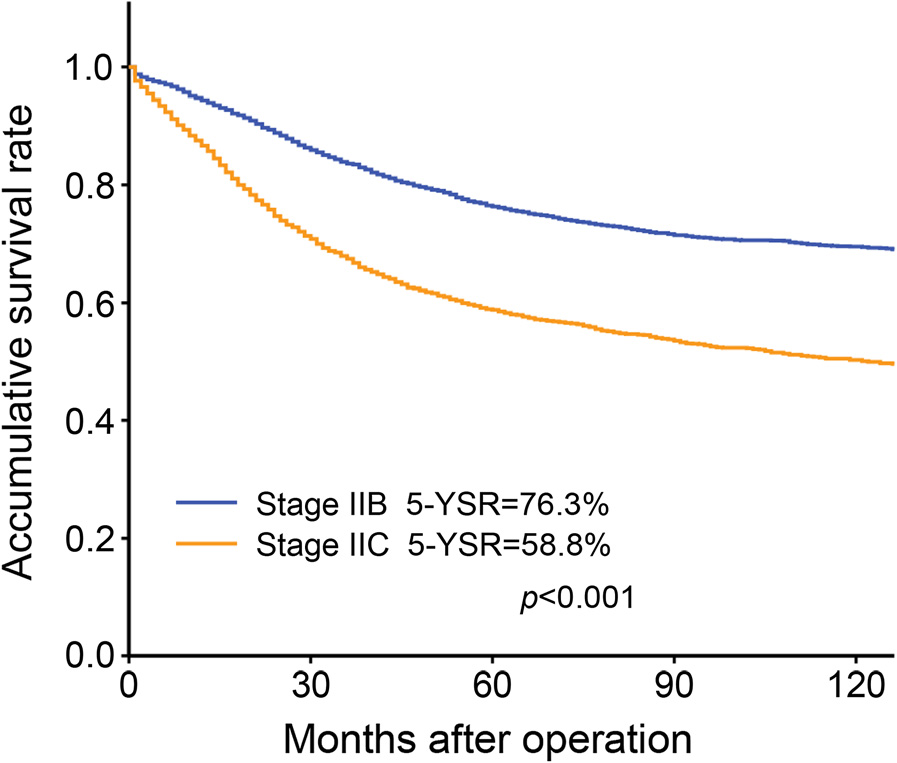
Differences in cause-specific survival between patients in stage IIB and stage IIC of the seventh edition of the TNM staging system.

**Figure 4:**
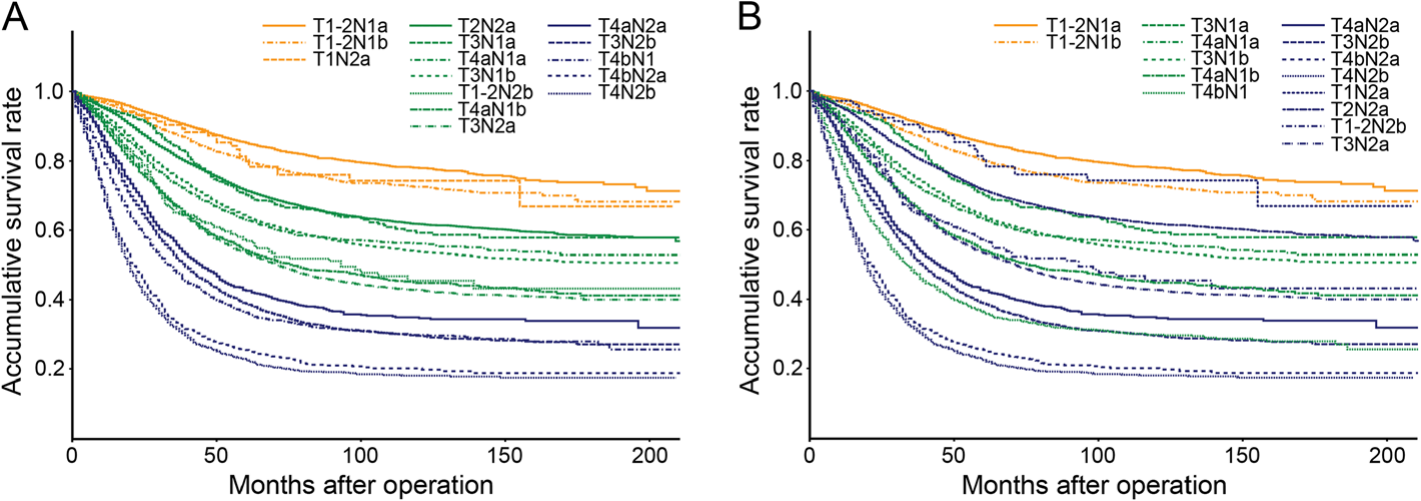
**Survival curves of patients in stage III.** Survival curves of patients in stage IIIA (yellow lines), IIIB (green lines), IIIC (blue lines) according to (**a**), seventh, or (**b**), sixth edition of the TNM staging system.

Revisions appeared in the AJCC-7 concerning stage II, stage III, and stage IV patients. Regarding stage II, we found significant differences in prognosis between patients categorized as AJCC-7 stage IIB and IIC (P < 0.001) (Figure 3). Regarding stage III, the patients with similar prognoses were sub-grouped into the same stage and there were significant differences in survival among patients in stage IIIA, IIIB, and IIIC (P < 0.001; Figure 4a). However, based on AJCC-6, some patients were not sub-grouped into a reasonable stage: 1) no significant difference in survival was found between patients with T1N2a in stage IIIC and patients with T1-2N1b in stage IIIA (P = 0.874); 2) there was no significant difference between patients with T2N2a in stage IIIC and patients with T3N1a in stage IIIB (P = 0.785); 3) the prognosis of patients with T4aN1b in stage IIIB was similar to patients with T1-2N2b in stage IIIC (P = 0.595) and patients with T3N2a in stage IIIC (P = 0.404; Figure 4b). These results support the revisions found in AJCC-7.

After the comparisons of prognoses among patients from the SEER dataset in different AJCC-6 stages stratified by AJCC-7, and among those in different AJCC-7 stages stratified by AJCC-6, we found that there were significant differences in survival for the patients in AJCC-6 stage IIB (P < 0.001), IIIB (P < 0.001), and IIIC (P < 0.001) when stratified by AJCC-7. When stratified by AJCC-6, significant differences in survival were not observed for AJCC-7 stage IIIA (P = 0.517) and IIIC (P = 0.283) patients; however, differences were observed for AJCC-7 stage IIIB (P < 0.001) patients (Table 2).

**Table 2:** Comparison of 5-year survival rate based on the 6th edition of TNM system according to the 7th edition of TNM system

	**The 7th edition (n, 5-YSR**^**b**^**)**								
	**I**	**IIA**	**IIB**	**IIC**	**IIIA**	**IIIB**	**IIIC**	**IV**	***P *value**
The 6th edition									
I	32464(92.5%)								N/A^c^
IIA		46635(83.6%)							N/A
IIB			4868(76.3%)	3323(58.8%)					<0.001
IIIA					5211(83.1%)				N/A
IIIB						25814(66.6%)	2002(36.3%)		<0.001
IIIC					108(80.4%)	8009(54.8%)	8686(35.0%)		<0.001
IV								21363(10.4%)	N/A
*P* value	N/A	N/A	N/A	N/A	0.517	<0.001	0.283	N/A	

5-YSR^b^: 5-year survival rate.

N/A^c^: not applicable.

### Comparison of the prognostic capacity between AJCC-7 and AJCC-6

Statistical assessment of the prognostic performance of the two editions by the Harrell’s c-index revealed a value of 0.7692 for AJCC-7, which was significantly better than 0.7663 for AJCC-6 (P < 0.001; Table 3). The prognostic performances of the two staging systems were also compared using BIC. As shown in Table 3, the prognostic performance of AJCC-7 was significantly superior to AJCC-6 (P < 0.001).

**Table 3:** Compare Harrell’s C index and Bayesian information criterion between the 6th and 7th edition TNM staging system

	**Harrell’s C index**			**BIC**^**a**^	
	**Coefficient**	**95% CI**^**b**^	***P *value**	**Coefficient**	***P *value**
			<0.001		<0.001
The 6th edition TNM stage	0.7663	0.7644-0.7683		−49847.26	
The 7th edition TNM stage	0.7692	0.7673-0.7712		−53817.48	

BIC^a^: Bayesian Information Criterion.

CI^b^: confidence interval.

## Discussion

Accurate staging is necessary to evaluate the prognosis of patients and is a critical element in determining appropriate treatment based on the experiences and outcomes of groups of prior patients with a similar stage. The AJCC TNM system is currently the most clinically useful staging system. However, unresolved issues in the latest edition remain and further investigation is warranted. Whether AJCC-7 exhibits a greater predictive capacity compared to AJCC-6 remains unclear [10,11,13]. For the first time, we compared the predictive capacity between AJCC-7 and AJCC-6 using two independent datasets.

Compared with AJCC-6, stage IIB is subdivided into IIB (T4aN0) and IIC (T4bN0) in AJCC-7. Lan [9] and Nitsche [13] proposed that there was not a marked difference in outcome between the two sub-stages. Kim [8] also found no significant difference in survival rate between patients with stage IIB and stage IIC cancer, but proposed that there was a trend toward a better survival rate for patients with stage IIB cancer. Based on the SEER dataset, a report by Gunderson indicated that there were large differences in the 5-year survival rate between patients with stage IIB and stage IIC cancer [6]. In the present study, we found a significant difference in prognosis between AJCC-7 stage IIB and IIC patients (P < 0.001, Figure 3). It is valuable to subdivide patients into IIB and IIC based on whether the tumor directly invades or is histologically adherent to other organs or structures.

For stage III patients, those who exhibited a similar prognosis were adequately sub-grouped into the same stage according to AJCC-7 (Figure 4a). However, based on AJCC-6, some patients were not sub-grouped into a reasonable stage (Figure 4b). Therefore, the results were more in accordance with AJCC-7.

Based on the SEER dataset, we found that there were significant differences in survival for AJCC-6 stage IIB, IIIB, and IIIC patients when stratified by AJCC-7. However, no significant differences in prognosis were observed for the AJCC-7 stage IIIA and IIIC patients when stratified by AJCC-6 (Table 2). Although there was a significant difference in survival for AJCC-7 stage IIIB when stratified by AJCC-6, the staging was reasonable because the difference in survival between the migrated patients and patients both in stage IIIC of AJCC-6 and AJCC-7 was even more significant (5-year survival rate: 54.8% vs. 34.0%, p < 0.001). These results indicated that using AJCC-7 could potentially provide more detailed classification and greater power to subgroup patients with a more homogenous prognosis compared to AJCC-6.

Two comprehensive statistical methods, Harrell’s C index and BIC, were used to compare the predictive capacity of AJCC-7 and AJCC-6. Both of the two tests revealed that the prognostic performance of AJCC-7 was significantly better compared to AJCC-6 (Table 3). Nitsche [13] proposed that AJCC-7 did not provide greater accuracy in predicting colorectal cancer patient prognosis compared with AJCC-6 based on a European single-center collective. However, in their published data we found that AJCC-7 exhibited a better predictive capacity according to the results of c-index and BIC, although the differences were not significant. It is possible that the differences between the results of the two tests were caused by different sample sizes. Stage migration occurred in only 8.48% (13442/158483) of patients from the SEER dataset used in this study and 13.5% (302/2229) of patients from the study by Nitsche. The total number of reclassified patients was small. Therefore, only when the comparison was based on a large dataset such as SEER the difference between AJCC-6 and AJCC-7 was significantly validated. When the comparisons were based on a relatively small dataset, it was difficult to obtain significant results, although a tendency that AJCC-7 exhibited a greater predictive capacity compared to AJCC-6 could be observed.

The classification of tumor deposits varied enormously in AJCC-7. These changes have been criticized as confusing and as exhibiting low reproducibility in the study by Nagtegaal [11]; however, they also proposed that AJCC-7 exhibited greater prognostic value than other staging systems when all tumor deposits irrespective of size or contour were included as lymph nodes. In our previous study [17], we found that the classification of tumor deposits in AJCC-7 satisfactorily predicted patients’ outcome for those without lymph node metastases. However, we also found that patients who were categorized as T3-4N2b with tumor deposits should be reclassified as stage IV. In the present study, it was not possible to compare the classification of tumor deposits in AJCC-7 with that in AJCC-6 because the SEER dataset did not identify the status of tumor deposits. Furthermore, the number of metastatic organ/site was unknown in the SEER program. Considering these two points, this study answers only well defined parts of the question about the comparison of AJCC-7 with AJCC-6.

Our study does have some limitations. It is a retrospective exploratory study. Clinical and pathologic patient information can be heterogeneous because SEER collects information from 12 population-based cancer registries. On the other hand, in the SEER dataset, data on adjuvant therapy is limited to information on radiation therapy only and the information of chemotherapy is unavailable.

## Conclusions

This study demonstrates that AJCC-7 is superior to the AJCC-6 staging system in predictive capacity. We recommend that this staging system be considered for clinical application.

## Consent

Written informed consent was obtained from the patient for publication of this report and any accompanying images.

## Abbreviations

AJCC: American Joint Committee on Cancer; SEER: National Cancer Institute’s Surveillance, Epidemiology, and End Results; BIC: Bayesian Information Criterion; ICD-O-3: International Classification of Diseases for Oncology, third edition; SD: Standard deviation; CI: Confidence interval; 5-YSR: 5-year survival rate.

## Competing interests

The authors declare that they have no competing interests.

## Authors’ contributions

PG and YS contributed equally to this work. PG, YS and ZW made a substantial contribution to study conception and design, statistical analysis, acquisition of data, and writing of the article. LT made a substantial contribution to acquisition of data. YX, JS, MY, and HX made significant contributions to the acquisition of data and writing of the article. All authors read and approved the final manuscript.

## Pre-publication history

The pre-publication history for this paper can be accessed here:

http://www.biomedcentral.com/1471-2407/13/123/prepub
